# The pregnant mouse uterus exhibits a functional kisspeptin/KISS1R signaling system on the day of embryo implantation

**DOI:** 10.1186/s12958-015-0105-1

**Published:** 2015-09-18

**Authors:** Mehri Fayazi, Michele Calder, Moshmi Bhattacharya, George A. Vilos, Stephen Power, Andy V. Babwah

**Affiliations:** The Children’s Health Research Institute, Victoria Research Laboratories, 800 Commissioners Road East, London, ON Canada N6C 2V5; Lawson Health Research Institute, London, ON Canada; Department of Obstetrics and Gynaecology, Division of Reproductive Endocrinology and Infertility, London, ON Canada N6C 2V5; Department of Physiology and Pharmacology, London, ON Canada N6C 2V5; Department of Oncology, London, Ontario University of Western Ontario, London, ON Canada N6C 2V5

**Keywords:** KISS1R, Kisspeptin, Uterus, Embryo, Implantation, p38, ERK1/2, SAPK/JNK, MAPK

## Abstract

**Background:**

Expression of kisspeptin (protein) and *Kiss1r* (mRNA) was recently documented in the mouse uterus on D4 of pregnancy (the day of embryo implantation) suggesting that the uterine-based kisspeptin (KP)/kisspeptin receptor (KISS1R) signaling system regulates embryo implantation. Despite this important suggestion, it was never demonstrated that the uterus actually exhibits a functional KP/KISS1R signaling system on D4 of pregnancy. Thus, the goal of this study was to determine whether a functional KP/KISS1R signaling system exists in the mouse uterus on D4 of pregnancy.

**Findings:**

Since kisspeptin/KISS1R signaling triggers the phosphorylation of the mitogen-activated protein kinases p38 and ERK1/2, through immunohistochemical analyses, we determined whether exogenously administered kisspeptin could trigger p38 and ERK1/2 phosphorylation in the uterus on D4 of pregnancy. The results clearly demonstrated that kisspeptin could and that its effects were mediated via KISS1R. Additionally, the robust kisspeptin-triggered response was observed in the pregnant uterus only. Finally, it was demonstrated that on D4 of pregnancy the *Kiss1* null uterus expresses functional KISS1R molecules capable of mediating the effects of kisspeptin.

**Conclusions:**

These results lead us to conclude that on D4 of pregnancy, the mouse uterus expresses a functional KP/KISS1R signaling system strengthening the possibility that this signaling system regulates embryo implantation. These findings strengthen the rationale for determining whether such a functional system exists in the uterus of the human female and if so, what role it might play in human pregnancy.

## Introduction

It is now well-established that hypothalamic kisspeptins (KPs), signaling via their cognate receptor KISS1R, are potent triggers of gonadotropin-releasing hormone (GnRH) secretion and mice bearing a deletion of the gene encoding KISS1R in the GnRH neuron display hypogonadotropic hypogonadism and infertility [1, 2]. Therefore, the hypothalamic-based KP/KISS1R signaling system is considered a major regulator of reproduction [3]. The KP/KISS1R signaling system is also expressed in the female reproductive tract and roles have been proposed in positively regulating follicular development, ovulation, embryo implantation and endometrial decidualization [4–7]. Therefore, the peripheral KP/KISS1R signaling system is considered a major regulator of reproduction [8].

We have previously reported that KP (protein) and *Kiss1r* (mRNA) are highly expressed in the mouse uterus on the day of implantation (that is, D4 after mating with the day of mating designated D0) suggesting a role for this signaling system in regulating embryo implantation [4]. To test this idea further, we turned our attention to mice bearing a global deletion of *Kiss1*. These mice are infertile and it was assumed that their infertility was due to a lack of hypothalamic KP/KISS1R signaling and the resulting reduction in gonadotropins, estradiol and progesterone [9]. However, despite the add-back of these hormones embryos failed to implant in the *Kiss1* null uterus leading us to suggest that it was a lack of uterine and not hypothalamic KP/KISS1R signaling that triggered implantation failure. Despite this important suggestion, we did not actually demonstrate the presence of a functional KP/KISS1R signaling system in the uterus on D4 of pregnancy. Such demonstration would have greatly strengthened our suggestion.

Thus, the goal of this study was to determine whether on D4 of pregnancy, the day of embryo implantation, a functional KP/KISS1R signaling system exists in the mouse uterus. It was previously reported that in various cell types, the KP/KISS1R signaling triggers mitogen-activated protein kinase (MAPK) phosphorylation. Specifically, it was demonstrated that KP treatment of KISS1R-expressing cells triggers the phosphorylation of the MAPKs p38 and ERK1/2 [10–12] but not SAPK/JNK [10]. We therefore investigated whether exogenously administered KP could trigger the phosphorylation of MAPKs in the uterus.

## Findings

In the following studies 8–12 week old 129S1/SvImJ female mice were used. WT females and estradiol- and gonadotropin-primed *Kiss1* null females (as previously described [4]) were mated to WT males (D0) and on the morning of D4 of pregnancy, females which displayed a copulatory plug (evidence of successful mating) were injected with either phosphate-buffered saline (PBS, 100 μl) or KP54 (100 nmol/kg, 100 μl, Tocris Bioscience, Minneapolis, MN, USA) intraperitoneally and 30 min later were rapidly sacrificed. In addition to the use of these pregnant mice, age-matched WT unmated but cycling females were also treated with PBS or KP54 in an identical manner. All experiments were repeated 6 independent times.

Immediately after drug or saline treatment, uteri were removed and simultaneously processed for immunohistochemistry using standard techniques [4] and 5 μm-thick slices were simultaneously immunostained to detect phosphorylated p38, ERK1/2 and SAPK/JNK. Care was taken to expose the experimental and control tissue to identical experimental conditions. Phosphorylated p38, pERK1/2 and SAPK/JNK were assayed using the following rabbit monoclonal antibodies: phospho-p38 (D3F9), phospho-p44/42 (ERK1/2) (D13.14.4E) and phospho-SAPK/JNK (81E11) (Cell Signaling, Boston, MA, USA). Antigen-bound primary antibodies were detected with the ImmunoCruz rabbit ABC Staining System sc-2018 (Santa Cruz Biotechnology, Inc. Dallas, TX, USA). Both the primary and secondary detection systems were used according to the manufacturers’ guidelines without any adaptations.

### KP triggers p38 phosphorylation in the uterine luminal and glandular epithelia on D4 of pregnancy

**Fig. 1:**
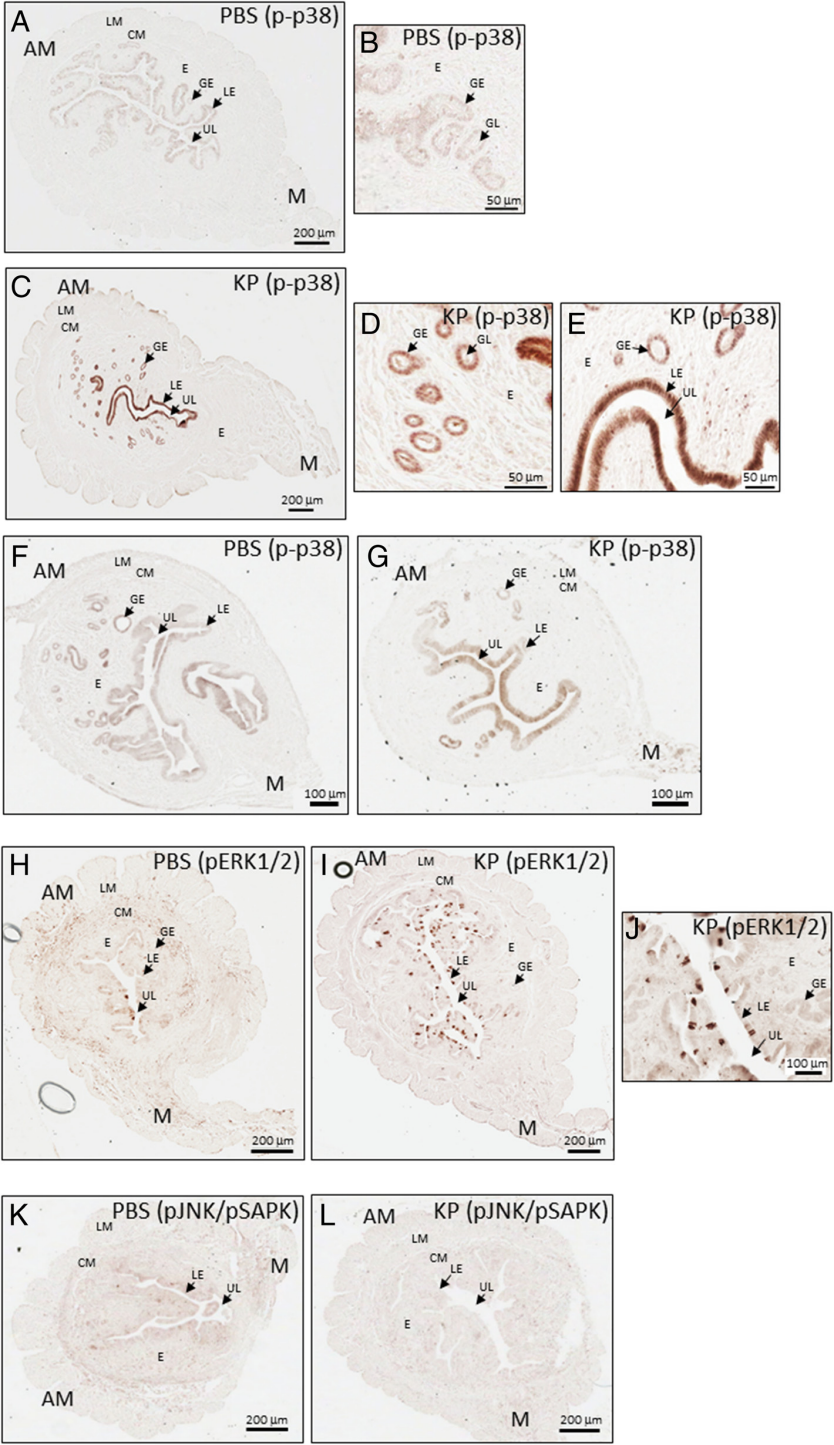
KP54 administration to female WT mice on D4 of pregnancy triggers p38 and ERK1/2 phosphorylation but not SAPK/JNK. Phosphorylated p38 expression in the D4 pregnant uterus following PBS-treatment (**a**); part of the uterus shown in (**a**) is magnified to better reveal details on the endometrial glands (**b**). Posphorylated p38 (p-p38) expression in the D4 pregnant uterus following KP54-treatment (**c**); parts of the uterus shown in (**c**) are magnified to better reveal details on the endometrial glands and luminal epithelium (**d** and **e**). Phosphorylated p38 expression in the non-pregnant uterus following PBS- (**f**) or KP54 (**g**) treatment. Phosphorylated ERK1/2 (pERK1/2) expression in the D4 pregnant uterus following PBS-treatment (**h**) or KP54-treatment (**i**); part of the uterus shown in (**i**) is magnified to better reveal details on the luminal epithelium (**j**). Phosphorylated SAPK/JNK (pSAPK/pJNK) expression in the D4 pregnant uterus following PBS-treatment (**k**) or KP54-treatment (**l**). AM: anti-mesometrial pole; LM: longitudinal muscle of the myometrium; CM: circular muscle of the myometrium; E: endometrium; LE: luminal epithelium; GE: glandular epithelium; M: mesometrial pole; UL: uterine lumen

Results showed that, compared to PBS-treated pregnant mice (Fig. 1a and b), uteri from KP54-treated pregnant mice exhibited (approximately 5-10-fold) higher levels of phosphorylated p38 in all cells of both the luminal and glandular epithelia (Fig. 1c, d and e). These findings were recapitulated when uteri were removed from untreated pregnant mice and then treated with either PBS or KP54 in vitro in the absence of any effects that KP54 might have triggered in vivo at the level of the hypothalamus, pituitary and ovaries (data not shown).

### KP barely triggers p38 phosphorylation in the non-pregnant uterus

When these studies were conducted in non-pregnant mice, relative to PBS-treated mice (Fig. 1f), uteri from KP54-treated mice (Fig. 1g) failed to show a strong increase in phosphorylated p38 in any cell type. However, careful examination of several uteri did suggest there was a very weak (approximately 1.2-fold greater) but almost undetectable response in the uterine epithelium. This suggest that KISS1R is weakly expressed in the non-pregnant uterus and/or poorly coupled to p38 phosphorylation. The weak expression suggests the possibility that basal kisspeptin signaling might also play a role in the non-pregnant uterus or that basal expression keeps the uterus in a primed-stated such that it allows for a more rapid upregulation of signaling when it is required during pregnancy.

### KP triggers ERK1/2 phosphorylation in the uterine luminal epithelium on D4 of pregnancy

When compared to PBS-treated pregnant mice (Fig. 1h), uteri from KP54-treated pregnant mice exhibited visibly higher levels of phosphorylated ERK1/2 in a subset of cells within the luminal epithelium only (Fig. 1i and j). When this study was conducted in non-pregnant mice, relative to PBS-treated mice, uteri from KP54 treated mice failed to show a visible increase in phosphorylated ERK1/2 in any cell type (data not shown) again supporting the idea that KISS1R is very weakly expressed in the non-pregnant uterus and/or uncoupled to ERK1/2 phosphorylation.

### KP fails to trigger SAPK/JNK phosphorylation in the uterus

Consistent with data obtained in KISS1R-expressing Chinese hamster ovary cells [10], relative to PBS-treated pregnant mice (Fig. 1k), uteri from KP54-treated pregnant mice (Fig. 1l) failed to show an increase in phosphorylated SAPK/JNK in any cell type. Failure to detect evidence of SAPK/JNK phosphorylation was not due to a loss in antibody quality since an aliquot of this antibody detected phosphorylated SAPK/JNK in UV-treated human embryonic kidney 293 cells (data not shown).

### KP triggers p38 phosphorylation in a KISS1R-specific manner in the uterus on D4 of pregnancy

It was reported that in addition to KISS1R, KP can signal via the NPFF1/GPR147 and NPFF2/GPR74 receptors [13]. Therefore, to determine whether the KP-triggered phosphorylation of p38 and ERK1/2 was KISS1R-specific the following experiment was conducted. Eight to 12 week old WT females were mated to WT males and on the morning of D4 of pregnancy, females were injected intraperitoneally with either PBS (100 μl) or the KISS1R antagonist P234 (100 nmol/kg, 100 μl, Tocris Bioscience, Minneapolis, MN, USA) [14]. Thirty minutes later a group of PBS-treated females was injected with either PBS (100 μl) or KP54 (100 nmol/kg, 100 μl) while the P234-treated group was injected with KP54 (100 nmol/kg, 100 μl). Thirty minutes later, mice were sacrificed and uteri were processed to assay for phosphorylated p38.

**Fig. 2:**
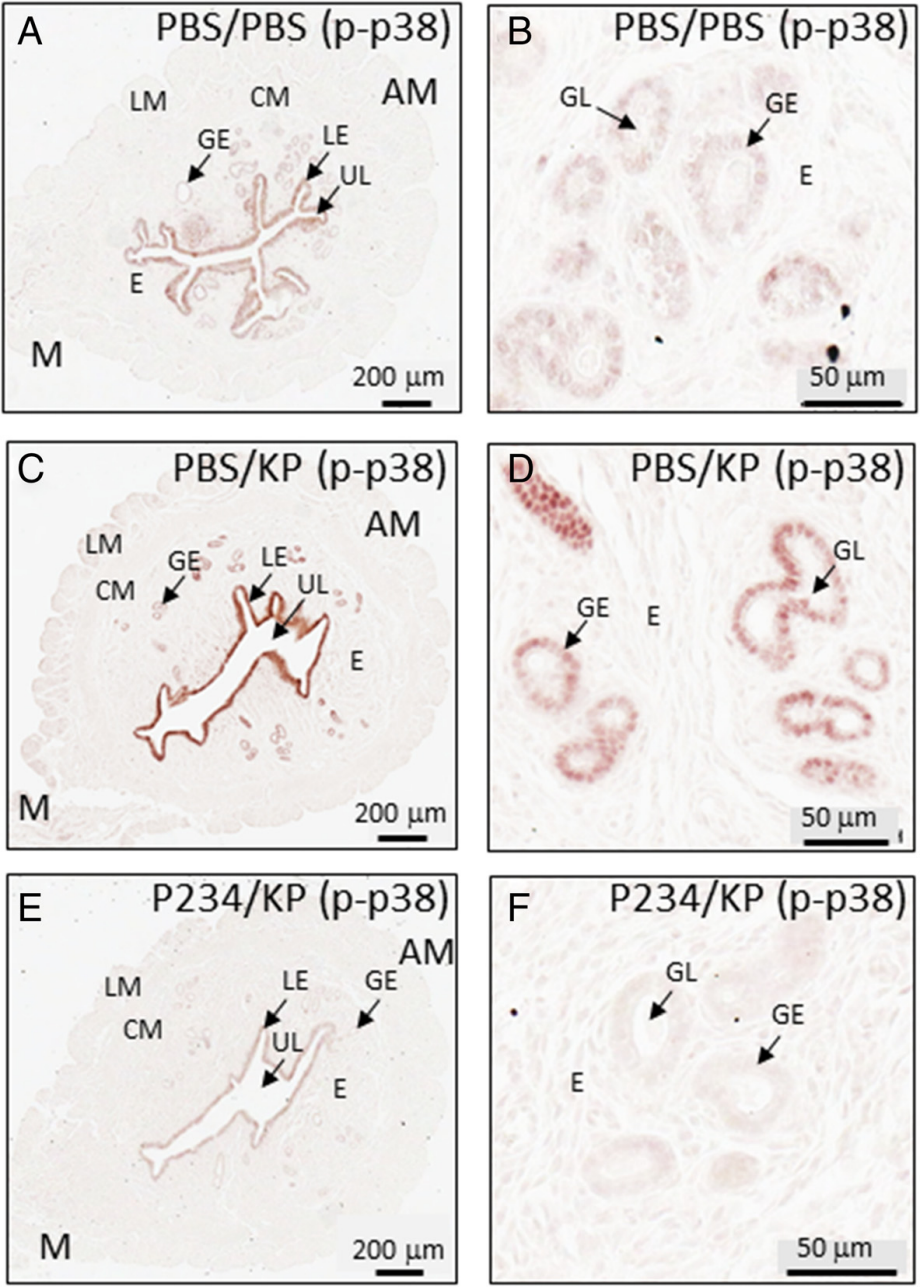
KP signals specifically via KISS1R in the uterus on D4 of pregnancy in WT mice. Phosphorylated p38 expression in the D4 pregnant uterus following 30 min PBS pre-treatment followed by 30 min PBS post-treatment (**a**); part of the uterus shown in (**a**) is magnified to better reveal details on the endometrial glands (**b**). Phosphorylated p38 expression in the D4 pregnant uterus following 30 min PBS pre-treatment followed by 30 min KP54 post-treatment (**c**); part of the uterus shown in (**c**) is magnified to better reveal details on the endometrial glands (**d**). Phosphorylated p38 expression in the D4 pregnant uterus following 30 min P234 pre-treatment followed by 30 min KP54 post-treatment (**e**); part of the uterus shown in (**e**) is magnified to better reveal details on the endometrial glands (**f**). AM: anti-mesometrial pole; LM: longitudinal muscle of the myometrium; CM: circular muscle of the myometrium; E: endometrium; LE: luminal epithelium; GE: glandular epithelium; M: mesometrial pole; UL: uterine lumen

As expected, based on earlier findings (Fig. 1a-e) results revealed that relative to PBS-treated pregnant mice (Fig. 2a and b), KP-triggered a robust increase in phosphorylated p38 levels in both the luminal and glandular epithelia of pregnant uteri (Fig. 2c and d), however, this KP-triggered increase was completely blocked when mice were pretreated with P234 (Fig. 2e and f). Interestingly, in P234 pretreated mice, phosphorylated p38 levels were visibly lower (by approximately 2-fold) than that of PBS only treated mice (Fig. 2e and f vs. 2a and b) suggesting that on the day of implantation, endogenous KP phosphorylates uterine p38. Based on preliminary data (*n* = 2), similar observations were made with pERK1/2 levels at the luminal epithelium and again, consistent with earlier experiments, KP54 had no effect on JNK/SAPK phosphorylated levels both in the presence and absence of P234 pre-treatment (*n* = 2) (data not shown).

### The *Kiss1*^−/−^ uterus remains responsive to KP

**Fig. 3:**
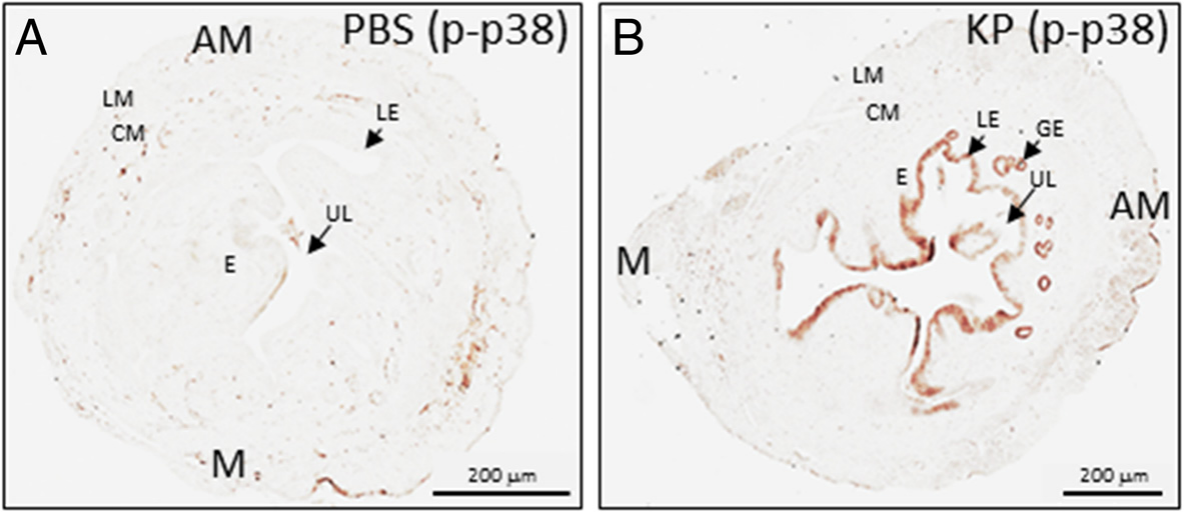
KP54 administration to female *Kiss1* ^−/−^ mice on D4 of pregnancy triggers p38 phosphorylation. Phosphorylated p38 expression in the D4 pregnant uterus following PBS-treatment (**a**) or KP54-treatment (**b**). AM: anti-mesometrial pole; LM: longitudinal muscle of the myometrium; CM: circular muscle of the myometrium; E: endometrium; LE: luminal epithelium; GE: glandular epithelium; M: mesometrial pole; UL: uterine lumen

In a previous study we failed to rescue the implantation defect through the add-back of KP to pregnant *Kiss1*^−/−^ females [4]. We speculated that this could have been due to the inefficient delivery of the drug (which was administered subcutaneously) to the uterus [15]. Another possibility was that although *Kiss1r* mRNA was still expressed in the *Kiss1*^−/−^ uterus, this expression did not result in the formation of functional receptor molecules thus precluding the ability of KP to signal and rescue the implantation defect. To test the latter possibility, *Kiss1*^−/−^ uteri on D4 of pregnancy were assayed for phosphorylated p38 following KP54 treatment. Results showed that relative to PBS-treated pregnant mice (Fig. 3a), uteri from KP54-treated pregnant mice exhibited higher levels of phosphorylated p38 in all cells of both the luminal and glandular epithelia (Fig. 3b). Based on preliminary data (*n* = 2), similar observations were made with pERK1/2 levels at the luminal epithelium and again, consistent with earlier experiments, KP54 had no effect on JNK/SAPK phosphorylated levels (*n* = 2) (data not shown). Together, these results confirm the presence of functional KISS1R molecules coupled to p38 and ERK1/2 phosphorylation on D4 of pregnancy in the *Kiss1*^−/−^ uterus suggesting that failure of exogenous KP to rescue the implantation defect, as reported by Calder et al. [4] was not due to the lack of functional KISS1R molecules in the uterus.

## Conclusions

The goal of this study was to determine whether on D4 of pregnancy, the day of embryo implantation, a functional KP/KISS1R signaling system exists in the mouse uterus. Our data clearly reveal that one exists and this major finding further strengthens the possibility that this signaling system regulates uterine functions in early pregnancy. Because expression, as determined by phosphorylated p38 and ERK1/2 levels was localized to the uterine epithelia, it is possible that KP/KISS1R signaling regulates embryo implantation. For example, through p38 it might regulate the availability (production and/or secretion) of glandular products essential for implantation, or through both p38 and ERK1/2 might regulate embryo implantation at the uterine epithelium. It is also possible that through paracrine signaling, epithelial KP/KISS1R signaling prepares stromal cells for decidualization in the event of successful implantation [7, 16].

## Abbreviations

KP: Kisspeptin
KISS1R: Kisspeptin receptor
GnRH: Gonadotropin-releasing hormone
MAPK: Mitogen-activated protein kinase
